# Shifted dynamic interactions between subcortical nuclei and inferior frontal gyri during response preparation in persistent developmental stuttering

**DOI:** 10.1007/s00429-017-1476-1

**Published:** 2017-07-24

**Authors:** F. Luise Metzger, Tibor Auer, Gunther Helms, Walter Paulus, Jens Frahm, Martin Sommer, Nicole E. Neef

**Affiliations:** 1 Department of Clinical Neurophysiology, Georg August University, Göttingen, Germany; 2 Biomedizinische NMR Forschungs GmbH am Max-Planck-Institut für Biophysikalische Chemie, Göttingen, Germany; 3 MRC Cognition and Brain Sciences Unit, Cambridge, UK; 4 Department of Psychology, Royal Holloway, University of London, Egham, UK; 5 Department of Medical Radiation Physics, Lund University, Lund, Sweden; 6 Department of Neuropsychology, Max Planck Institute for Human Cognitive and Brain Sciences, Stephanstraße 1a, 04103 Leipzig, Germany

**Keywords:** Persistent developmental stuttering, Substantia nigra, Response anticipation, Basal ganglia, Inferior frontal gyrus, Disinhibition

## Abstract

Persistent developmental stuttering is associated with basal ganglia dysfunction or dopamine dysregulation. Here, we studied whole-brain functional connectivity to test how basal ganglia structures coordinate and reorganize sensorimotor brain networks in stuttering. To this end, adults who stutter and fluent speakers (control participants) performed a response anticipation paradigm in the MRI scanner. The preparation of a manual Go/No-Go response reliably produced activity in the basal ganglia and thalamus and particularly in the substantia nigra. Strikingly, in adults who stutter, substantia nigra activity correlated positively with stuttering severity. Furthermore, functional connectivity analyses yielded altered task-related network formations in adults who stutter compared to fluent speakers. Specifically, in adults who stutter, the globus pallidus and the thalamus showed increased network synchronization with the inferior frontal gyrus. This implies dynamic shifts in the response preparation-related network organization through the basal ganglia in the context of a non-speech motor task in stuttering. Here we discuss current findings in the traditional framework of how D1 and D2 receptor activity shapes focused movement selection, thereby suggesting a disproportional involvement of the direct and the indirect pathway in stuttering.

**Electronic supplementary material:**

The online version of this article (doi:10.1007/s00429-017-1476-1) contains supplementary material, which is available to authorized users.

## Introduction

Persistent stuttering is a neurodevelopmental speech fluency disorder characterized by involuntary speech blocks, sound and syllable repetitions, and sound prolongations. The onset of stuttering occurs most often between the ages of three and six, affects more than 5% of children, and manifests in 0.72% of the adult population, predominantly in males (Yairi and Ambrose 1999; Craig 2002; Howell et al. 2008; Yairi and Ambrose 2013). Stuttering phenotypes are diverse, life-span history varies across subjects, and degree of severity spans the whole spectrum from very mild to very severe. Participation in communication can be largely restricted. Resulting emotional and socio-economic consequences can seriously compromise quality of life.

Aetiology and pathogenesis of persistent developmental stuttering are still obscure (Bloodstein and Ratner 2008). Over the last few decades, a huge body of literature has provided cumulating evidence for irregular neurophysiological signs of the trait of stuttering. Several neurobiological characterizations have been described that are tightly related to each other and that shape the neurophysiological understanding of stuttering. Consistent reports of an imbalanced cortical lateralization during speech tasks manifest the idea of an aberrant hemispheric specialization (Orton and Travis 1929; Travis 1978; Foundas et al. 2001). Two recent quantitative reviews on neuroimaging studies robustly confirmed the imbalanced activation patterns associated with speech production in persistent developmental stuttering (Budde et al. 2014; Belyk et al. 2015). According to these and a previous ALE meta-analyses (Brown et al. 2005), the neural signatures of stuttering are characterized by overactivation of the cerebellum and of right frontal motor regions including the premotor cortex, inferior frontal gyrus, insula, and the operculum and underactivation of the auditory cortex. These often-replicated findings are based on group statistics. In contrast, case-study approaches represent the heterogeneous patterns that emerge due to compensation through one’s lifetime, and different types of treatment. Accordingly, case studies provide additional valuable insights into the complex neural architecture of stuttering (Ingham et al. 2012; Wymbs et al. 2013). Diffusion magnetic resonance imaging (dMRI) repeatedly provided evidence for less coherent white matter structures (Sommer et al. 2002; Chang et al. 2008, 2015; Watkins et al. 2008; Kell et al. 2009; Cykowski et al. 2010; Kronfeld-Duenias et al. 2014, 2016). Affected connections might impede the signal transfer between language-related and speech-related left fronto-parieto-temporal brain regions as summarized in a recent quantitative review (Neef et al. 2015a). Fluent speech production evolves from dynamic network organizations, but functional connectivity within these networks is aberrant in those who stutter (Lu et al. 2009, 2010a, b; Chang et al. 2011; Chang and Zhu 2013). The spatio-temporal patterning, and particularly the timing of neuronal signals guiding fluent speech production, is not sufficiently tuned (Kent 2000; Salmelin et al. 2000; Ludlow and Loucks 2003; Alm 2004; Etchell et al. 2014). Inhibitory and excitatory intracortical circuits of the ventral primary motor cortex exhibit a reduced dynamic range possibly restricting the proper encoding of ongoing, competing speech motor programs (Neef et al. 2011b, 2015b; Neef 2013). A dysfunction of the basal ganglia circuits or a dysregulation of the dopamine system (Wu et al. 1995, 1997; Braun et al. 1997; Alm 2004; Giraud et al. 2008) might be related to an imprecise cortical input to the striatum and result in an inappropriate excitation of the motor cortex or left inferior frontal/ventral premotor regions. Neurofunctional signs of persistent stuttering are not only restricted to speech movements, but also affect the non-speech motor system (Chang et al. 2009; Neef et al. 2011a; Markett et al. 2016), indicating a broad implication of sensorimotor brain circuits.

The gradient order directions into velocities of articulators (GODIVA) model is a neurocomputational model that provides a mechanistic understanding of speech motor control (Guenther 1995; Guenther et al. 1998; Bohland et al. 2009). The model utilizes a feedforward and a feedback control system to simulate activity across connected brain regions, which results in learning and producing words. An extended version of the GODIVA model showed that both a disconnection of cortico-striatal pathways as well as a dysregulation of the dopamine system resulted in stuttering (Civier et al. 2013). Simulated neural irregularities caused a delayed readout of the motor program via affected basal ganglia thalamo–cortical circuits. This integrated framework of speech production suggested that in the context of stuttering an aberrant timing of neural signalling closely relates to a dysfunction of the basal ganglia, which comprises an erratic excitation and inhibition of engaged neuronal populations.

Numerous clinical studies support the notion that a dysfunction of the basal ganglia is involved in persistent developmental stuttering. Direct evidence for basal ganglia involvement comes from studies with deep brain stimulation in clinical populations. In patients with Parkinson’s disease or primary dystonia, for example, stimulation of the subthalamic nucleus, globus pallidus internus, or ventral intermediate nucleus of the thalamus induces stuttering or modulates preoperative comorbid stuttering: either aggravating or ameliorating it (Burghaus et al. 2005; Nebel et al. 2009; Walker et al. 2009; Allert et al. 2010; Toft and Dietrichs 2011; Risch et al. 2015). The occurrence of basal ganglia disorders, such as Parkinson’s disease, often leads to a re-emergence of recovered developmental stuttering (Shahed and Jankovic 2001). Acquired stuttering after brain injury is associated with lesions in the thalamus or striatum (Lundgren et al. 2010). Furthermore, functional neuroimaging in adults with persistent developmental stuttering repeatedly showed altered activity of the basal ganglia during speech tasks. In adults who stutter, stuttered reading is associated with increased activity in the left globus pallidus and left lateral thalamus as compared to fluent reading (Fox et al. 1996). Speaking under normal or altered auditory feedback is associated with an overactivation of a broad cluster in the midbrain of adults who stutter compared to control participants; strongest activity was found in the substantia nigra encompassing also the pedunculopontine nucleus, subthalamic nucleus, and red nucleus, as well as the left and right posterior lobes of the cerebellum (Watkins et al. 2008). Stuttering therapy caused an increase of activity in the red nucleus (Neumann et al. 2003). During a reading task, activity of the caudate nucleus correlated positively with stuttering severity, while a negative correlation between activity in the substantia nigra and degree of stuttering severity is reported for both pre- and post-treatment (Giraud et al. 2008). Altered functional connectivity between basal ganglia and cortical regions has been observed (Lu et al. 2009, 2010a, b; Chang and Zhu 2013), and disturbed structural connectivity between cortical and subcortical regions has also been reported (Watkins et al. 2008; Connally et al. 2014; Chang et al. 2015).

The idea of a dysregulation of the dopamine system in persistent developmental stuttering finds support in studies using positron emission tomography (PET). Thereby, the distribution of dopamine receptors can be visualized for the specific engagement of brain regions in certain tasks. Speech production caused an increased uptake of 6-FDOPA in the left caudate tail, left pulvinar, right hypothalamus, medial prefrontal cortex, deep orbital cortex, insular cortex, and auditory cortex, suggesting excessive dopaminergic activity in involved brain regions in adults with persistent developmental stuttering (Wu et al. 1997). Additional support for a hyperdopaminergic state in developmental stuttering comes from the effect of dopamine and dopamine receptor effectors. While levodopa, converted to dopamine, worsens speech fluency (Anderson et al. 1999), dopamine antagonists, such as haloperidol, risperidone, or olanzapine, typically improve speech fluency (Lavid et al. 1999; Maguire et al. 2004). However, the use of pharmacological agents for the treatment of stuttering is currently under debate (Bothe et al. 2008; Boyd et al. 2011) because of provoked adverse side effects (Maguire et al. 2004). Despite the described dopaminergic directionality of the effect in persistent developmental stuttering, in Parkinson’s disease stuttering-like dysfluencies can be related to both increased and decreased dopamine levels (Goberman and Blomgren 2003). Thus, basal ganglia dysfunction in persistent developmental stuttering remains to be established more directly, and the nature of a possible dysregulation in the cortico–striato–cortical loop is yet to be characterized (Giraud et al. 2008).

The circuitry connecting the cortex and the basal ganglia comprises multiple parallel cortico-striatal input and striatonigral output systems (Gerfen 1984). The substantia nigra pars compacta (SNc) is one of the core basal ganglia substrates of dopamine synthesis containing a massive accumulation of dopaminergic neurons densely modulating striatal activity (Dahlstroem and Fuxe 1964; Felten and Sladek 1983). The substantia nigra pars reticularis (SNr) mostly consists of inhibitory GABAergic neurons (Tepper and Lee 2007). In models of cortico-basal ganglia circuits, SNc/SNr constitute a complex nonlinearly operating linchpin, conveying direct, indirect, and hyperdirect inputs (Alexander and Crutcher 1990; Mink 1996; Swanson 2000; Nambu et al. 2002). Generated output enables the selection of appropriate motor action such as speaking. Dopaminergic neurons as well as GABAergic neurons of the SNc/SNr receive (1) direct inhibitory input from cortico-striatal fibres; (2) indirect excitatory input via cortico–striato–pallidal synaptic transmissions through the subthalamic nucleus; (3) hyperdirect excitatory transsynaptic input via cortico–subthalamic nucleus projections, and (4) cortical input from the somatosensory cortex and the motor cortex (Watabe-Uchida et al. 2012). Together with the internal segment of the globus pallidus (GPi), SNr is a main output nucleus of the basal ganglia. Projections target thalamic and brainstem nuclei that further project to a broad range of cortical areas (Deniau et al. 2007).

Given the complexity and massive connectivity of basal ganglia circuits and its nonlinear dynamics on the organization of functional network activity, it is difficult to infer mechanistic principles by means of functional magnetic resonance imaging (fMRI). It is important to consider that multiple factors constrain the interpretation of blood oxygenation level-dependent (BOLD) responses (Düzel et al. 2009). BOLD responses indicate changes in the concentration of deoxyhaemoglobin in the vicinity of red blood cells and vessels, a physiological process caused by increased blood flow, volume, and oxygenation, and which accompanies neural activity (Bandettini et al. 1994). Neurophysiological investigations associate BOLD responses with input and intracortical operations rather than output processing (Logothetis et al. 2001). Neuromodulatory transmitters, such as dopamine regulate processes in neural circuits, but their effect on BOLD is still under investigation (Zaldivar et al. 2014).

To improve the understanding of how neurotransmitter producing substrates contribute to the formation and organization of functional neural networks across the whole human neocortex, it is necessary to draw inferences from noninvasive neuroimaging studies in humans. One feasible way is to study the functional connectivity of dopaminergic nuclei. Resting-state network architecture and task-state architecture are closely matched to each other, indicating that such analyses reflect an intrinsic standard architecture of functional brain organization (Cole et al. 2014). For the substantia nigra, connectivity analyses of fMRI data are rather scarce and no study exists on the functional connectivity of the substantia nigra in stuttering. Only one study has reported psychophysiological interactions (PPI) of the SN/VTA (ventral tegmental area) in healthy adults. This study was related to cognitive control demands in a Stroop task (Köhler et al. 2016). The authors associated the functional connectivity between the SN and dorsal striatum, thalamus, supplementary motor area (SMA), and dorsal anterior cingulate cortex (ACC) with resolving the task-related motor conflict; functional connectivity between VTA and the ventral striatum and perigenual ACC was associated with goal-directed motivational processes. In addition, there is strong resting-state functional connectivity (RSFC) between the SN and cortical and subcortical regions (Tomasi and Volkow 2014; Murty et al. 2014; Zhang et al. 2016; Bianciardi et al. 2016; Bär et al. 2016). Cortical regions involve the dorsomedial frontal, somatomotor, superior temporal, inferior parietal, and occipital cortices (Murty et al. 2014), as well as regions of the default mode network such as the dorsal ACC and the posterior cingulate gyrus/precuneus (Bär et al. 2016). Subcortical connectivity involves the hippocampal complex, nucleus accumbens, putamen, globus pallidus, mediodorsal nucleus of the thalamus, lower brainstem, and several cerebellar nuclei. The current study examined whole-brain functional connectivity dynamics of the substantia nigra to better understand how this central hub coordinates and reorganizes sensorimotor brain networks in persistent developmental stuttering. To achieve this we employed an fMRI paradigm that reliably induces activity in the substantia nigra as shown by a previous study (Lütcke et al. 2008). Accordingly, we used a continuous performance test (CPT, van Leeuwen et al. 1998; Heinrich et al. 2004). During this task the presentation of a particular stimulus leads to the simultaneous anticipation and preparation of a Go/No-Go response within a predictable time interval. Electrophysiological studies suggest that the underlying anticipation process is related to a characteristic slow cortical potential termed contingent negative variation (Walter et al. 1964). It has been suggested that contingent negative variation engages an ensemble of basal ganglia–thalamo–cortical structures (Birbaumer et al. 1990), and electrophysiological studies in stuttering report its irregularities (Prescott and Andrews 1984; Prescott 1988; Walla et al. 2004; Vanhoutte et al. 2015, 2016). Lütcke et al. (2008) studied healthy participants and elucidated distinct brain networks of the early and the late component of the contingent negative variation (CNV), which typically evolve when carrying out the CPT with long inter-stimulus intervals (Loveless and Sanford 1974; Birbaumer et al. 1990). The early component is assumed to reflect an orientation reaction, while the late component might indicate the motor preparation (Rohrbaugh et al. 1976). The early component was associated with increased BOLD activity in the striatum, SMA, left motor cortex, and right premotor cortex (Lütcke et al. 2008), which might reflect the coordination of input information to the basal ganglia. The late component was associated with increased BOLD activity in the anterior cingulate cortex (ACC), the right fronto-polar cortex, bilateral insula, putamen, thalamus, and the substantia nigra (Lütcke et al. 2008) and thus might reflect the coordination of basal ganglia output. In this study, we aimed to explore whether the coordination of cortico–striato–nigral circuits characterizes the trait of stuttering. We decided to focus on the SN because it is the main source of dopamine synthesis for the motor and non-motor system and because stuttering is associated with a hyperdopaminergic state. Therefore, we planned a functional connectivity analysis, a psychophysiological interaction (PPI), which relies on a correlation analysis of a continuous regression variable subsuming the physiological time varying signal change of the substantia nigra and the time variance of the critical task condition. To gain robust PPI results, we decided not to distinguish between the early and the late component, but considered the whole time span of response anticipation, which included three subsequent brain volumes and thus time varying signals covering 6 s. For the random-effects analysis across PPI maps, we expected an activation map reflecting both cortico-striatal input and nigrostriatal output operations. Ultimately, group contrast maps were calculated to uncover possible deficiencies associated with stuttering. Results allowed us to discuss current data in a framework that accounts for a deficient motor preparation in stuttering, which is possibly related to an insufficient frontoparietal coupling mediated by corticostriatal–striatonigral brain networks.

## Materials and methods

### Participants

We examined 13 adults who stutter (AWS), 4 females, mean age 29.8 years, SEM 8.6 years, and 14 matched fluent-speaking participants, 5 females, mean age 27.4 years, SEM 6.0 years. All participants were right-handed according to the Edinburgh handedness inventory (Oldfield 1971). They were all native German speakers, except for one Hungarian and one bilingual Turkish–German, both AWS. Groups were matched for age, handedness, and years of formal education (1 = school; 2 = high school; 3 = less than 2 years college; 4 = 2 years college; 5 = 4 years college; 6 = postgraduate). None of the fluent speakers reported a family history of speech or language disorders. All participants were free of neurological or medical disorders or drug use that would potentially affect their neurological function based on self-report. They had normal structural MRI scans as confirmed by radiologists. Two of the participants who stutter were undergoing behavioural therapy at the time of participation in the study. None of the AWS was under pharmacological treatment. All subjects provided written informed consent prior to inclusion in the study. Ethical approval from the local ethical committee at the University Medical Center Goettingen and written, informed consent were obtained prior to the investigation. Subjects were paid 20 Euros for participating. Participants were recruited via advertisements, but those two AWS who underwent therapy were from the Institute of the Kassel Stuttering Therapy in Bad Emstal, Germany. All AWS reported to have participated in therapies throughout their lives. Only two participants were undergoing therapy during the time of the experiment. Because therapies were heterogonous in terms of approach (stuttering modification, fluency shaping, van Riper, etc.), time (during childhood or adulthood), and frequency, we did not consider therapy as a regression variable in statistical analysis. Table 1 summarizes demographic characteristics of the studied groups.

**Table 1:** Participants' demographic information and behavioural results

	Control	Adults who stutter	Difference
*N*	14	13	
Age in years (mean ± SD)	27.4 (6.0)	29.8 (8.6)	0.409^t^
Sex (male/female)	9/5	9/4	0.847^C^
Handedness [LQ, median (range)]	93 (60–100)	100 (77–100)	0.458^U^
Education [median (range)]	4 (2–6)	5 (2–6)	0.257^M^
% stuttered syllables [median (range)]	0.2 (0.1–0.9)	4.3 (3.1–62.4)	<0.001^U^
SSI-4 overall score [median (range)]	–	25 (16–48)	–

*SSI-4* stuttering severity instrument, third edition, *% stuttered disfluencies* stuttered syllables occurring per 100 syllables, *LQ* laterality quotient

^t^Group differences were tested by unpaired *t* test, or ^C^ Chi-squared test, ^U^ Mann–Whitney U test, or ^M^ independent samples median test

Stuttering severity was assessed by collecting samples of reading aloud and spontaneous speech elicited through a standardized interview asking participants to narrate their daily routine, to retell their favourite movie or novella, and to give directions when imagining a person asking the way. All participants spoke German, except the Hungarian (AWS), who spoke Hungarian. These samples were video-recorded and analysed offline by a qualified speech–language pathologist. The stuttering severity index (SSI-4) was employed to determine the frequency and duration of stuttered syllables as well as physical concomitants of stuttering (Riley 2008). According to SSI-4, five participants showed very mild stuttering, one was mild, three were moderate, and four were very severe. Supplementary Table 1 lists detailed information on individual characteristics.

### CPT task

**Fig. 1:**
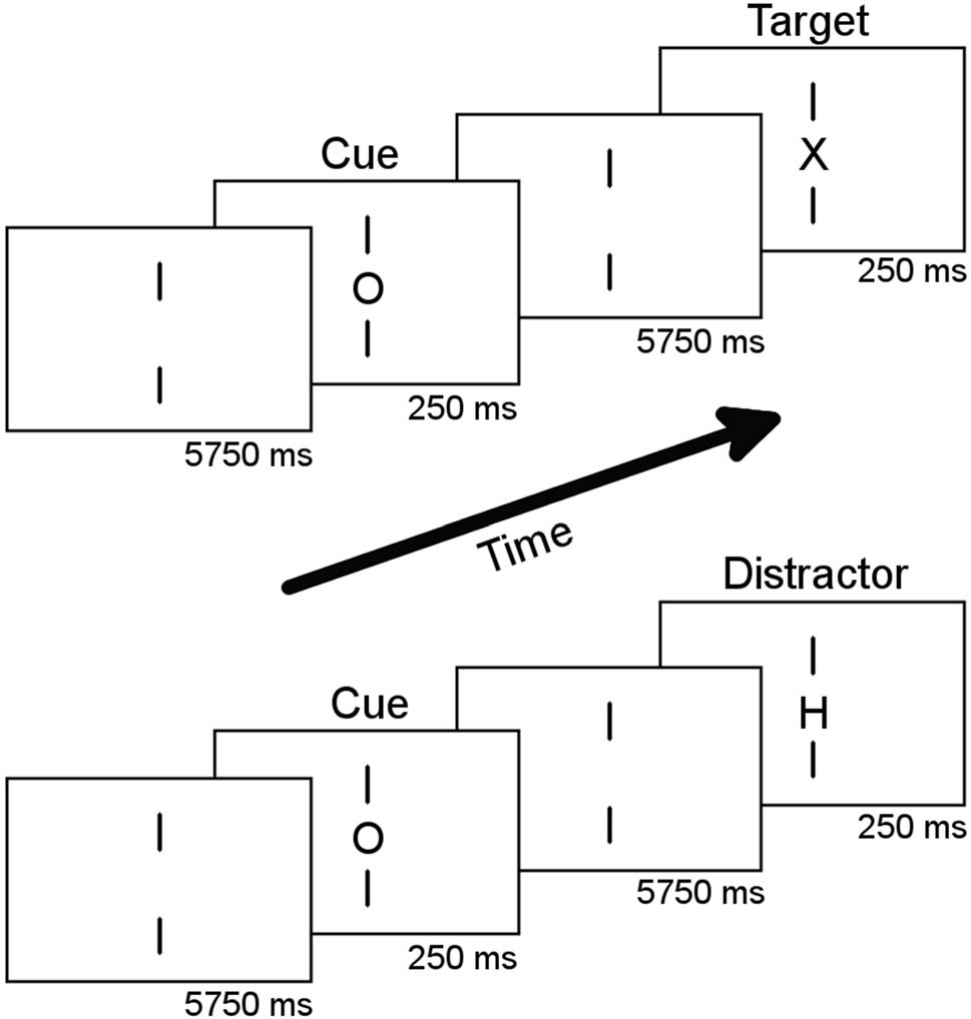
Schematic illustration of the continuous performance task. The three letters “*H*”, “*O*”, or “*X*” served as stimuli. Participants were asked to respond with a right-hand button press if the cue letter (*O*) was followed by the target letter (*X*). If the cue letter (*O*) was followed by any other letter (*H*, *O*), participants had to suppress the prepared motor response. The design was adopted from Lütcke et al. (2008)

Figure 1 gives a schematic representation of the continuous performance task (CPT) as conducted in the scanner. We employed a cued version of the CPT that has been shown to reliably elicit an activation of the substantia nigra in the response anticipation phase (Lütcke et al. 2008). Participants were presented with the letters O, X, or H. The stimuli were shown very briefly for 250 ms with an interstimulus interval of 5750 ms. Participants were instructed to press the response button as fast as possible with their right index finger if the cue letter O was followed by the target letter X (cue–target trial). If the cue letter O was followed by the distractor letter H (cue–distractor trial), participants were instructed not to respond. The distractor letter H could also be represented in place of a cue, signalling to the participants that any subsequent letter is irrelevant. Two vertical lines were continuously presented above and below the stimulus location to direct subjects’ attention to the centre of the screen. Participants were familiarized with the task outside the scanner and performed four runs of the CPT inside the scanner. Each run included 16 cue–target trials, 16 cue–distractor trials, and 16 uncued trials in a randomized fashion. The probability of occurrence of the cue letter O was 40%, H and X occurred with a probability of 30%, respectively. The duration of a run was 8 min and 8 s followed by a short break. The whole behavioural task lasted approximately 40 min. The choice of a hand-motor response instead of an oral-motor response holds the advantage of avoiding physiological artefacts that occur with speech movements in the MRI scanner (Callan et al. 2006), which might be further enhanced in AWS due to stuttering or additional head movements.

### Magnetic resonance imaging

Magnetic resonance imaging was conducted at 3 T (Siemens Tim Trio, Erlangen, Germany) using a 12-channel head coil. Structural whole-brain *T*_1_-weighted MRI for anatomical referencing was acquired using a non-selective inversion recovery 3D FLASH sequence (TR = 2530 ms, TE = 3.65 ms, flip angle = 7°, TI = 1100 ms) at a spatial resolution of 1.3 × 1 × 1.3 mm^3^. All fMRI measurements were based on a gradient-echo EPI sequence (TR = 2000 ms, TE = 36 ms, flip angle 70°, 244 volume per run) with 2 × 2 × 4 mm^3^ spatial resolution (96 × 96 acquisition matrix, 192 mm FOV, 7/8 partial Fourier phase encoding, bandwidth 1336 Hz/pixel, echo spacing 0.81 ms). We acquired 22 interleaved axial slices without any interslice gap, positioned roughly parallel to the intercommissural plane, covering the whole cerebrum. All images were corrected for motion in *k* space as supplied by the manufacturer (Siemens Healthcare, Erlangen, Germany). These motion-corrected images were used for analysis. At the end of each session, one EPI volume was acquired with the same specifications as the functional series, but covering the whole brain (36 slices) to facilitate registration of fMRI data to the T1-weighted image.

### Pre-processing and whole-brain fMRI analysis of response anticipation

FMRI data processing was carried out using FEAT version 6.0, a tool from the FMRIB Software library (FSL; http://fsl.fmrib.ox.ac.uk). Pre-processing involved an off-line motion correction by image-based registration (Jenkinson et al. 2002), smoothing with a Gaussian kernel of 5 mm full width at half maximum. Non-brain tissue was removed (Smith 2002) and all volumes were intensity-normalized by the same factor. Temporal high-pass filtering was achieved by Gaussian-weighted least-squares straight line fitting, with high-pass filter cut-off at 100 s. Because the fMRI datasets only covered part of the brain, a three-stage linear (FLIRT) and nonlinear (FNIRT) registration (Jenkinson and Smith 2001) was performed to register the partial-volume images via the whole-brain images and the anatomical T1-weighted 3D images into standard MNI space (Jenkinson and Smith 2001). The intersection mask of the final group analysis is displayed in Supplementary Fig. 1, illustrating that portions of the inferior temporal, occipital lobe, and cerebellum were not considered in the following group analyses. Boxcar models were convolved with a Gamma function. Model fit was determined by statistical time-series analysis in the framework of the general linear model. Omissions and false alarms were modelled as additional regression variables. The temporal derivative of the convolved waveform was added to take possible delays of the BOLD signal change into account. A within-subjects contrast between cue (O) and control trial (uncued H and uncued X) was calculated with a fixed-effect analysis. The duration of the cue O as well as the control trials (uncued H and uncued X) was modelled to be 6 s. Across participants, mixed-effects group analyses were calculated. *Z* (Gaussianised T/F) statistic images were thresholded using clusters determined by *Z* > 3.1 and a (FWE-corrected) cluster significance threshold of *p* = 0.05 (Worsley et al. 1996, Worsley 2001). Figure 3 shows the resulting activation maps that indicate the network of response anticipation.

### Definition of the substantia nigra

**Fig. 2:**
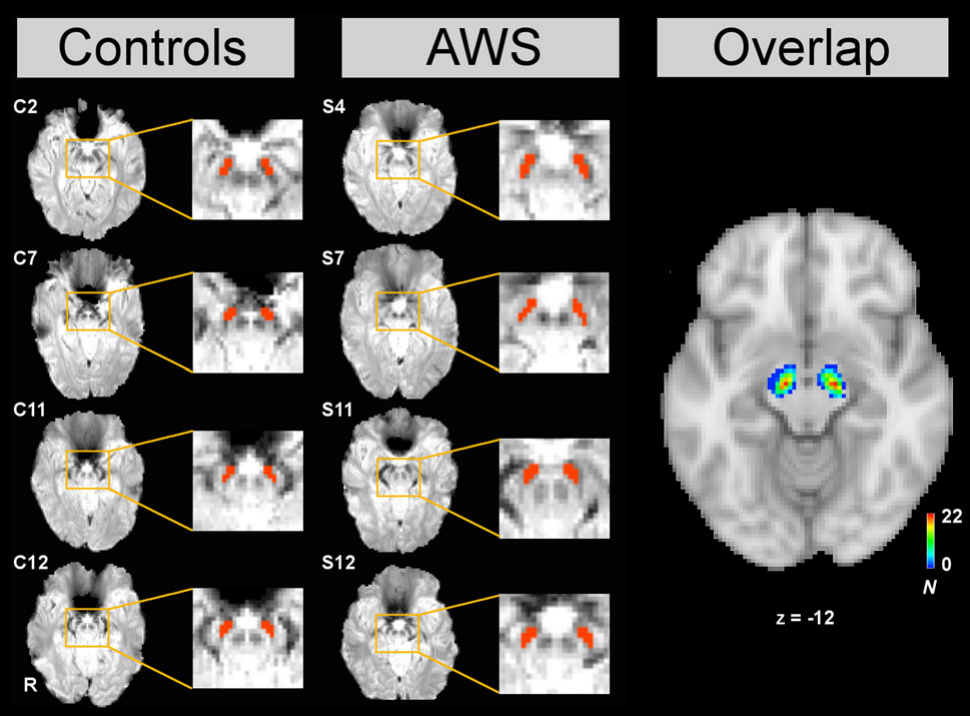
Definition of the seed region in the substantia nigra (SN). To extract physiological time courses of the SN, bilateral masks were manually drawn in the mean EPIs of every run of each participant. Four examples are displayed for control participants and adults who stutter (AWS), respectively. *Right columns* display the contrast of the mean EPI, *left rows* display SN masks overlaid in* red*. The axial brain slice in the *right column of the figure* illustrates the overlap of substantia nigra seed regions in the current data set across all 27 participants, aligned with the 2-mm MNI standard brain

The substantia nigra and the nucleus ruber are clearly distinguishable from surrounding structures in EPI images as illustrated in Fig. 2. The good contrast results from different tissue properties, that is, varying iron content and myelin content yielding different *T*_2_ or *T*_2_* relaxation times and different magnetic susceptibilities (Schweser et al. 2011). Anatomical seed masks were drawn manually for each participant and for each run using the FSL 4.1.4 viewer (http://fsl.fmrib.ox.ac.uk/fsl/fslwiki/). The first author of this manuscript carried out the segmentation using the following steps. First, a slice was determined that best represented the substantia nigra and that was at least two slices above the lowest slice of the field of view to avoid contamination due to motion artefacts in voxels at the border of the field of view. Second, the contrast values in the viewer for the image were set to maximally increase visibility of the substantia nigra. The contrast values were determined on an individual subject basis, and contrast values were kept constant between hemispheres. Third, the axial view was picked to delineate eight voxels in the substantia nigra in each hemisphere. Individual seed masks are shown in Fig. 2 for 8 representative participants. The decision to delineate 8 voxels 128 mm^3^ in the centre of the substantia nigra was reasonable because the substantia nigra (SNr/SNc) has a volume of about 281 mm^3^ (Plantinga et al. 2016), as quantified in a human post-mortem brain specimen. Analyses of in vivo data estimated the volume to be about 225 mm^3^ (Keuken et al. 2014). Translating this to the spatial resolution of the data presented in the current study, the substantia nigra should be covered by approximately 16 voxels. However, due to the applied spatial smoothing (5-mm FWHM), contamination of the signal by neighbouring structures such as the subthalamic nucleus or the ventral tegmental area is likely (de Hollander et al. 2015).

To compare the localization of the substantia nigra with previous reports, masks were registered to the MNI space employing the transformation matrix generated during the registration of the EPIs to the standard brain. The mean centre of gravity of the masks of AWS was [*x* = −11, *y* = −14, *z* = −11] for the left SN and [*x* = 13, *y* = −14, *z* = −12] for the right SN. Control subjects showed a centre of gravity at [*x* = −10, *y* = −14, *z* = −12] for the left SN and at [*x* = 12, *y* = −13, *z* = −12] for the right SN. All coordinates are in accordance with the reported location of the SN (de Hollander et al. 2015). Subjects in the two groups did not differ in the distribution of the seed coordinates. This was calculated by 2 × 3 repeated measures ANOVAS with coordinate as within-subjects factor and Group as between-subjects factor yielding *F*(1,25) = 0.562, *p* = 0.460 for the left substantia nigra, and *F*(1,25) = 0.898, *p* = 0.342 for the right substantia nigra. Table 2 summarizes the group statistics of the location of the substantia nigra. Analysis of variance was carried out with SPSS (IBM).

**Table 2:** Centre of gravity of the substantia nigra in the MNI brain

	Control	AWS	Effect of group
Left SN			
*x*	−10.09 (1.25)	−11.36 (1.78)	*F*(1,25) = 0.562 *p* = 0.460
*y*	−13.70 (1.44)	−14.23 (2.21)	
*z*	−11.71 (1.70)	− 10.77 (1.90)	
Right SN			
*x*	11.99 (1.39)	13.14 (1.81)	*F*(1,25) = 0.898 *p* = 0.342
*y*	−13.46 (1.63)	−14.16 (2.12)	
*z*	−11.76 (1.77)	−11.12 (2.05)	

### Region of interest analyses

The main anatomical region of interest (ROI) of the current study is the SN because of its substantial role in dopaminergic neurotransmission and the hypothesized hyperdopaminergic state in stuttering. SN ROI masks were derived from the manual segmentation of the individual SN voxels. We added two additional structures to the ROI analyses, the thalamus and the external segment of the globus pallidus (GPe). The anatomical ROI mask of the thalamus was constrained to the medial dorsal nucleus (MD) because this subregion showed the peak activation during response anticipation (Lütcke et al. 2008). ROI masks of GPe and MD were derived from the conversion of the original Talairach structural labelling atlas to the 2-mm MNI template (Lancaster et al. 2000, 2007). Parameter estimates were extracted from the cope images of the first-level contrasts for response anticipation via custom written shell scripts. For every subject, four values were extracted per mask. Extracted values were averaged and SPSS (IBM) was used to calculate statistics. Analyses of co-variance were calculated to test group effects, and Spearman rank correlations were calculated to test whether stuttering severity correlated with BOLD activity.

### Psychophysiological interaction analysis

To test whether coupling between ROIs and task-related brain regions was different between groups, we performed psychophysiological interaction (PPI) analyses (Friston et al. 1997). The PPI analyses were again carried out using FEAT version 6.0 (FSL; http://fsl.fmrib.ox.ac.uk). To this end, the time courses of the signal change in the left SN, left GPe, and left MD were extracted. For the SN we used the manually segmented individual masks, and for MD and GPe we used the conversion of the original Talairach structural labelling atlas to the 2-mm MNI template. The regression model of the whole brain task (see above) was extended by a physiological term (time course) and an interaction term. Specifically, for every run, one PPI analysis was calculated considering the product of the modelled cue (O) and the time course of the ROI as the regression variable modelling the PPI. The four contrast images resulting from the four runs of a given participant were averaged via fixed-effects analysis. Group comparisons were then calculated at higher level via random-effects analysis. Clusters were determined by *Z* > 2.3 and a corrected cluster significance threshold of *p* = 0.05 (Worsley 2001).

## Results

### No group differences in the continuous performance test

**Fig. 3:**
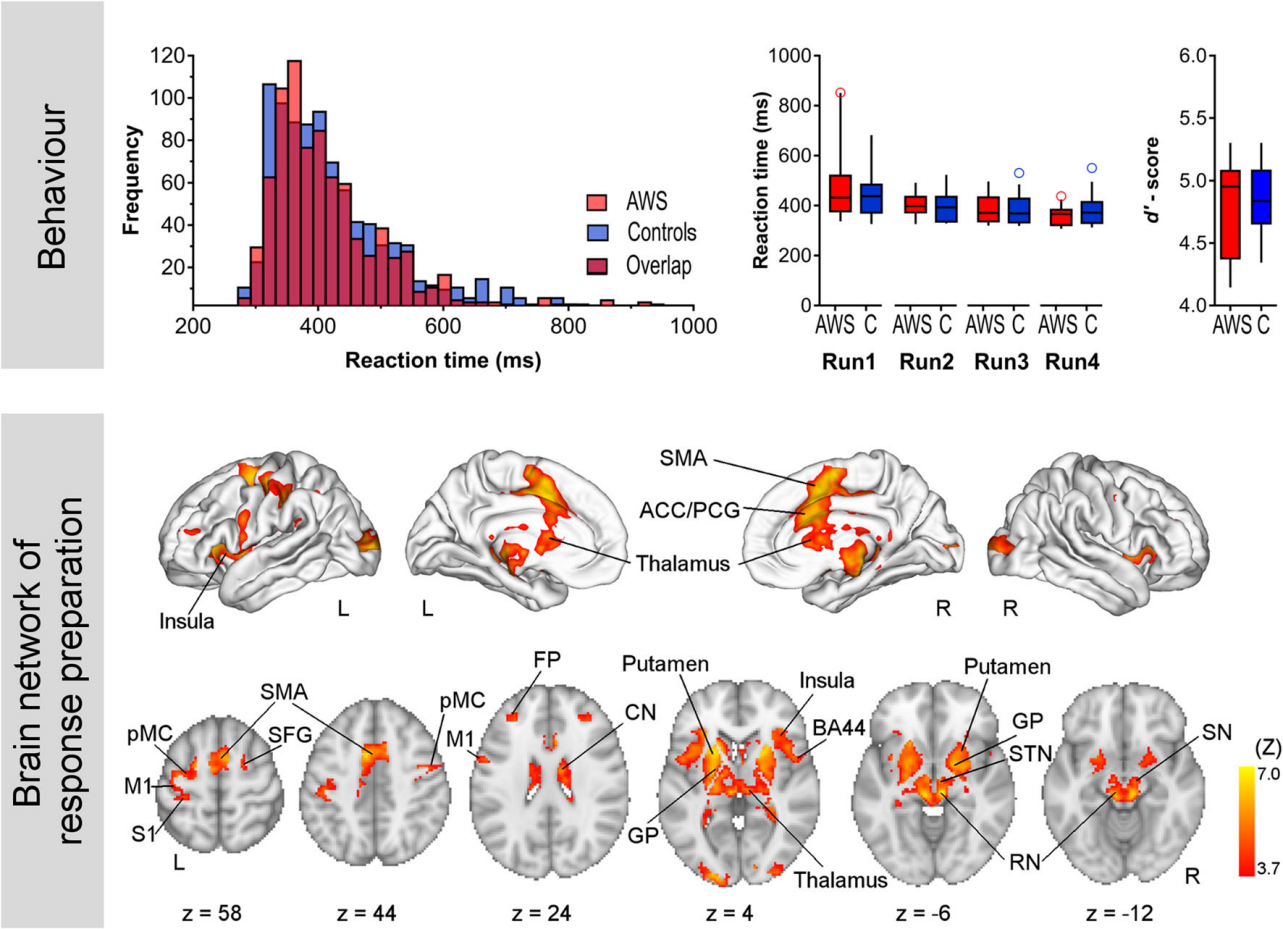
Results of the CPT. The *upper panel* histograms display all reaction times, separated for groups illustrating a broad overlap. For reaction times, box plots were calculated across median reaction times, separated for group and run. For accuracy, box plots were calculated across the pooled data. Both groups performed comparably well and only showed a few false alarms and omissions, resulting in high *d*′ scores. Whiskers display the 10th and 90th percentile. Maps in the *middle panel* show brain activations during response anticipation. The contrast was obtained by the comparison of activity following cues and non-cues across all participants. Anticipatory processes activated a large-scale cortical network including the frontal pole (FP), superior frontal gyrus (SFG), SMA, insula, inferior frontal gyrus pars opercularis (BA44), premotor cortex (pMC), motor cortex (M1), and somatosensory cortex (S1) together with the paracingulate gyrus (PCG) and anterior cingulate cortex (ACC). The broad activation of the basal ganglia and brainstem involving the caudate nucleus (CN), putamen, globus pallidus (GP), thalamus, red nucleus (RN), subthalamic nucleus (STN) and also the substantia nigra (SN) is most prominent. *Z* statistic images were thresholded using clusters determined by *Z* > 3.1 and a (corrected) cluster significance threshold of *p* = 0.05 (Worsley 2001)

On average, participants detected 98.75% of targets with a mean false alarm rate of 0.85%. No differences were found between AWS and control participants for hit rates [*U* = 80.5, *p*(27) = 0.616], false alarms [*U* = 63, *p*(27) = 0.616], or the combined measure *d*′ [*U* = 86.5, *p*(27) = 0.83]. The detection rate was at ceiling, indicating that the task was quite easy. Mean median reaction times were nominally shorter for AWS compared to control participants (Supplementary Table 2). However, nonparametric statistics revealed no group difference for reaction times. Histograms across pooled data showed a strong overlap of groups ensuring that both groups performed with comparable reaction times in the scanner (Fig. 3).

### Motor preparation recruits cortico–basal ganglia–thalamo–cortical loops

Response anticipation recruited large-scale brain networks in both study groups. Because the mixed-effects group analysis revealed no differences between groups, we show the corresponding activation map across all participants in Fig. 3 and report corresponding MNI coordinates in Supplementary Table 3. Cortical fronto-parietal structures involved are the frontal pole (FP), superior frontal gyrus (SFG), SMA, insula, inferior frontal gyrus pars opercularis (BA44), premotor cortex (pMC), motor cortex (M1), and somatosensory cortex (S1) together with the paracingulate gyrus (PCG) and the anterior cingulate gyrus (ACC). A massive bilateral subcortical activation of the basal ganglia and brainstem included the caudate nucleus (CN), putamen, globus pallidus (GP), thalamus, red nucleus (RN), subthalamic nucleus (STN) and also the substantia nigra (SN). Supplementary Fig. 2 illustrates task-positive brain activations separated for groups.

### Substantia nigra activity during response anticipation scales with stuttering severity

**Fig. 4:**
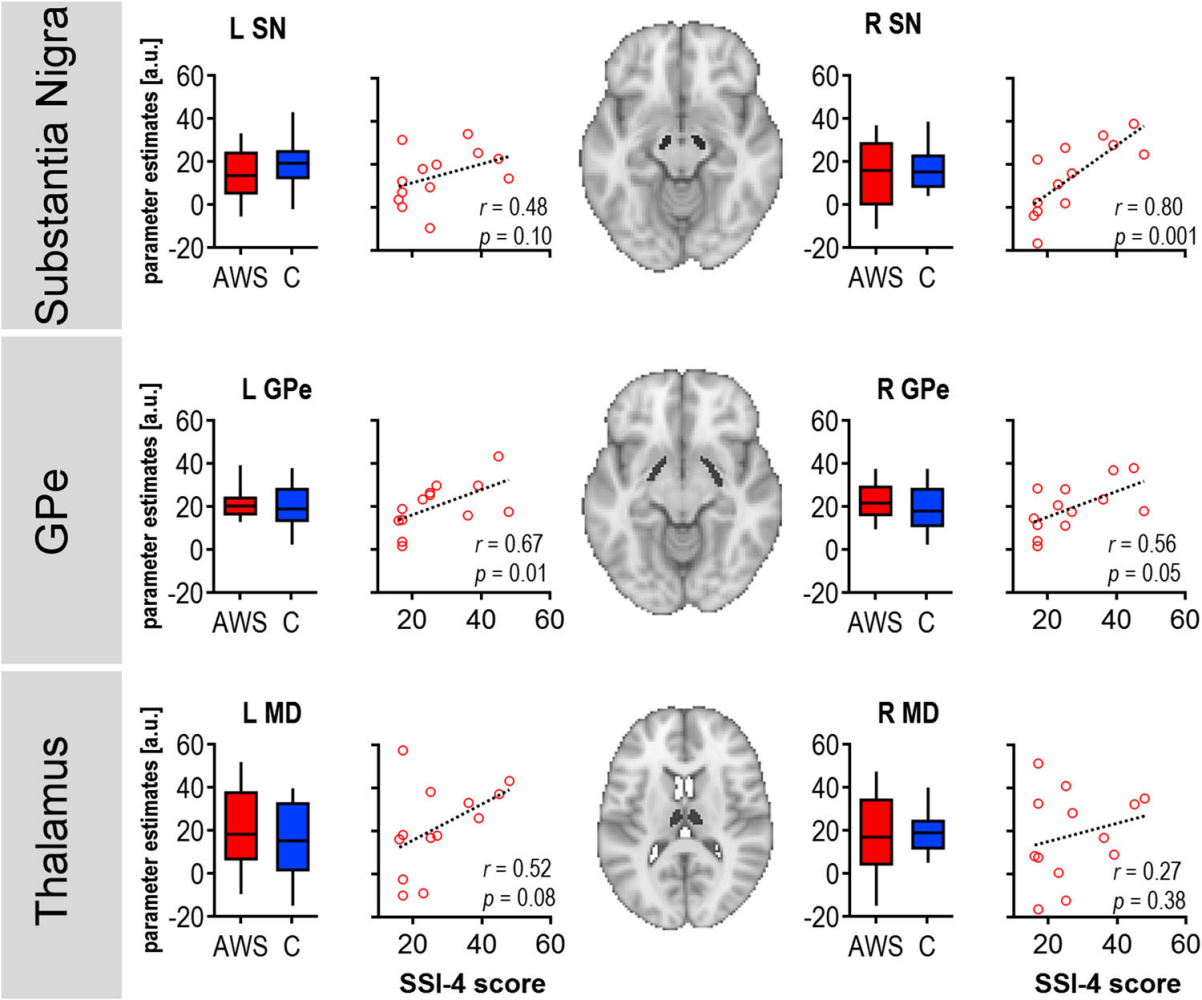
Region of interest analysis. The axial sections display the locations of the ROI masks of the substantia nigra (SN), external segment of the globus pallidus (GPe), and medial dorsal nucleus of the thalamus (MD). Box plots illustrate the BOLD activity during response anticipation separated for adults who stutter and control participants. Whiskers display the 10th and 90th percentile. Scatter plots show the relationship between BOLD activity and stuttering severity. Stuttering severity as indicated by the SSI-4 score was correlated positively with the task-related activation of the right SN. Marker sizes in the scatter plots indicate percent stuttered syllables with largest diameters for highest frequencies

ROI analyses confirmed the strong activation of the substantia nigra, MD, and GPe during response anticipation. Similarly to the whole-brain analysis, box plots in Fig. 4 also illustrate comparable activation levels in both groups. In AWS, stuttering severity was correlated positively with right substantia nigra activity [*r*_sp-SSI_ = 0.795, *p*(13) = 0.001, *r*_sp-%SS_ = 0.796, *p*(13) = 0.001]. This brain–behaviour relationship is illustrated in the scatter plot of Fig. 4. No further correlation was significant at the threshold of *p* < 0.008 set after Bonferroni correction for multiple testing.

### Functional connectivity of the SN

PPI yielded no significant results when seeding in the left SN. The uncorrected contrast map of all participants showed one cluster, located in the right premotor cortex at *x* = 32, *y* = −8, *z* = 56, with *Z* > 2.3, and *k* = 92.

### Functional connectivity of the MD and GPe

**Fig. 5:**
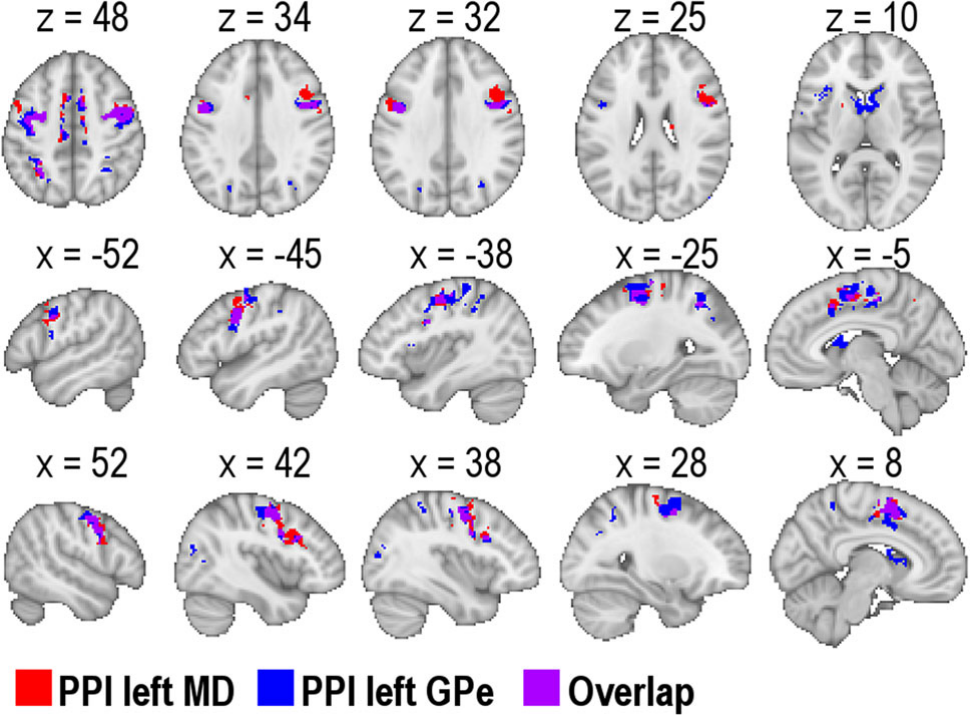
PPI results across adults who stutter and control participants during response anticipation seeding in the medial dorsal nucleus of the left thalamus (MD) and the external segment of the globus pallidus (GPe). *Z* statistic images were thresholded using clusters determined by *Z* > 2.3 and a corrected cluster significance threshold of *p* = 0.05 (Worsley 2001)

The left MD of the thalamus showed, bilaterally, large-scale functional connectivity with the caudate nucleus, premotor cortex, and SMA, additionally involving the right paracingulate gyrus, and the left superior frontal gyrus, left middle frontal gyrus, left anterior intra-parietal sulcus, and left superior parietal lobule (Table 3). This network is displayed on axial and sagittal brain slices in Fig. 5.

**Table 3:** Functional connectivity of the MD during response anticipation (*Z* > 2.3, *p* < 0.05)

Region	App BA	*x*	*y*	*z*	Peak *Z*	Voxels
L premotor cortex	6	−24	−6	48	4.45	1139
L superior frontal gyrus		−24	0	56	4.29	
L middle frontal gyrus		−26	−4	56	4.28	
L premotor cortex	6	−44	−10	50	4.26	
R premotor cortex	6	40	−2	48	4.89	1043
R premotor cortex	6	26	−6	48	4.67	
R supplementary motor area	6	8	0	52	4.21	875
R paracingulate gyrus		6	10	54	4.1	
L supplementary motor area	6	−6	−2	54	3.74	
L anterior intra-parietal sulcus	hIP3	−26	−54	46	3.51	299
L superior parietal lobule	7PC	−36	−48	52	3.25	
L superior parietal lobule	7A	−26	−60	52	3.16	
L caudate		−6	10	12	3.05	267
R caudate		6	14	10	3.01

The external segment of the left globus pallidus shows bilateral functional connectivity with the premotor cortex and the paracingulate cortex, additionally involving the left SMA, left superior frontal gyrus and the pars opercularis of the right inferior frontal gyrus (Table 4; Fig. 5).

**Table 4:** Functional connectivity of the GPe during response anticipation (*Z* > 2.3, *p* < 0.05)

Region	App BA	*x*	*y*	*z*	Peak *Z*	Voxels
R premotor cortex	6	52	4	38	4.36	771
R inferior frontal gyrus	44	54	6	26	3.72	
L premotor cortex	6	−18	−16	66	4.07	599
L superior frontal gyrus		−24	−6	58	3.66	
L supplementary motor area	6	−6	−6	52	4.15	435
R paracingulate gyrus		10	10	50	3.43	
L paracingulate gyrus		−6	12	48	3.37	

### PPI group differences

**Fig. 6:**
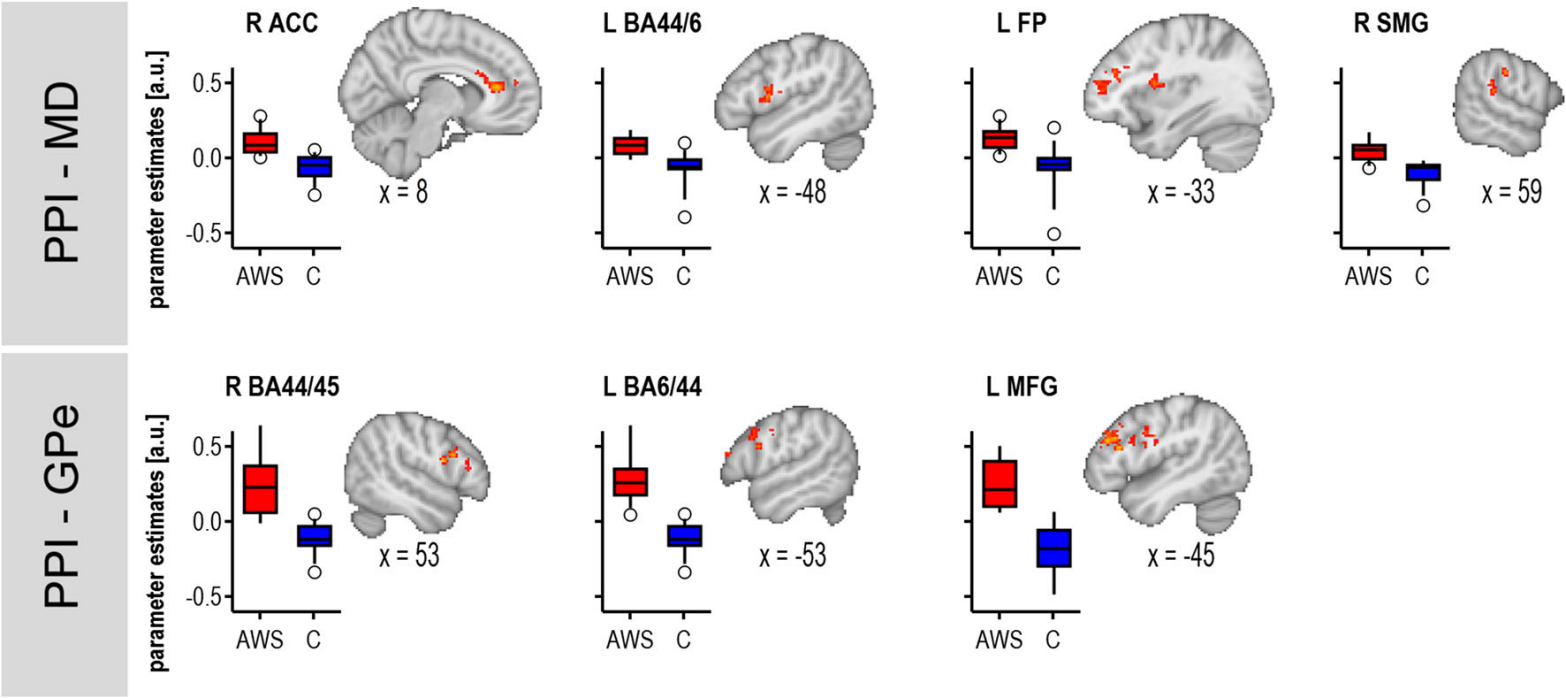
PPIs resulted in group differences when seeding in the medial dorsal nucleus of the left thalamus (MD) and when seeding in the external segment of the globus pallidus (GPe). *Z* statistic images were thresholded using clusters determined by *Z* > 2.3 and a corrected cluster significance threshold of *p* = 0.05 (Worsley 2001)

PPI group comparisons revealed different network dynamics during response anticipation for AWS compared to fluent speakers. Both seeding in the left MD and seeding in the left GPe revealed a psychophysiological interaction with the left inferior frontal gyrus pars opercularis (BA 44). This interaction was of an opposing direction. While AWS showed a positive PPI, fluent speakers showed a negative PPI. Similar PPI group differences occurred for the left MD in the left frontal pole, and in the right cingulate gyrus, anterior division (ACC) and right supramarginal gyrus (SMG, also labelled as inferior parietal lobule, Caspers et al. 2013). For the left GPe further group differences involved the left middle frontal gyrus and the right inferior frontal gyrus pars triangularis (BA 45) as well as BA44. All PPI group differences are displayed in Fig. 6 and summarized in Supplementary Tables 4 and 5.

## Discussion

During the anticipation of a motor response, the neural system heavily involves subcortical nuclei including the SN. Because the SN is a core neural substrate of dopamine synthesis and because persistent developmental stuttering is associated with a hyperdopaminergic state, the SN constituted the main region of interest in the current fMRI study. Our first main finding is the positive correlation between the severity of stuttering and task-related activity in the SN. This correlation implies that the SN is a core neural hub of this speech fluency disorder. Furthermore, our second major finding shows that the task-related dynamical network formation with the GPe, an upstream nucleus of the indirect pathway, is different in AWS compared to fluent speakers. Because the GPe is a principal nucleus of the indirect pathway, our second major finding relates a different dynamic synchronization of fronto–basal ganglia–thalamo–cortical networks during the preparation of a motor response to an altered implementation of D2 receptor-mediated functions. The following discussion elaborates on these two novel findings, differentially relating brain function to brain regions crucial for persistent developmental stuttering.

### Positive correlation between stuttering severity and SN activity

The current study shows that severe stuttering relates to higher SN activity during response anticipation. This positive relationship is compatible with the suggested hyperdopaminergic state in stuttering. In this regard, increased SN activity might reflect increased release of dopamine in the striatum via nigrostriatal dopaminergic neurons. This needs to be interpreted with caution because the relationship between dopaminergic neurotransmission and BOLD response is not yet clear (Zaldivar et al. 2014). Nevertheless, the observed brain–behaviour relationship is in line with previous reports: adults who stutter show excessive firing of the SN (Wu et al. 1995; Watkins et al. 2008), as well as, a disproportionate release of dopamine in the striatum (Wu et al. 1997).

The new aspect of our finding is the context in which this correlation occurred, a continuous performance task. Previous neuroimaging studies associated increased SN activity with brain activity during speech tasks. For example, reading aloud was shown to yield higher SN activity in AWS compared to fluent speakers (Watkins et al. 2008). Moreover, stuttering severity was shown to correlate with SN activity (Giraud et al. 2008). In contrast, the current study showed that a positive correlation between stuttering severity and SN activity relates to the anticipation and preparation of a manual motor response. This finding is in line with neuroimaging studies that report altered brain activity during the preparation or planning of speech motor responses in AWS compared to fluent speakers (Salmelin et al. 2000; Chang et al. 2009; Vanhoutte et al. 2015; Mock et al. 2015, 2016). In the context of a basal ganglia disorder, studies of the CNV are of special interest because the generators of the late CNV are most likely the basal ganglia (Ikeda et al. 1997; Bareš and Rektor 2001). Recent studies on stuttering with a CNV task report an increased CNV slope in AWS compared to fluent speakers (Vanhoutte et al. 2015) and a negative correlation between stuttering rate and event-related desynchronization of alpha and beta band activity (Mock et al. 2016). Beta desynchronization during motor preparation likely reflects the suppression of intrinsic rhythms of the motor cortex activation (Crone et al. 1998; Pfurtscheller and Lopes da Silva 1999). Thus, diminished suppression in severe cases of AWS as reported by Mock et al. (2016) is highly plausible. Postulated theories on the function of beta band activity also support the plausibility of this observation. It has been proposed that beta band activity is the idling rhythm in the motor system and that a pathological enhancement, as evident during stuttering, hinders flexible switching between current state and upcoming behavioural and cognitive states (Engel and Fries 2010). More direct evidence for the CNV to be associated with SN functioning comes from studies of patients with Parkinson’s disease. The characteristic neuropathological sign of Morbus Parkinson is the degeneration of dopaminergic neurons in the SNc. Patients with Parkinson’s disease show a reduced amplitude of the late CNV (Praamstra et al. 1996) and an attenuated preparatory desynchronization of alpha and beta band oscillatory activity (Praamstra and Pope 2007). Accordingly, observations of altered electrophysiological signs during CNV tasks in AWS support the view that in stuttering the preparation of a motor response is associated with altered basal ganglia activity. Moreover, dopaminergic SNc neurons are most likely involved.

Our data prove the contribution of the SN to the cognitive state of response anticipation and preparation. However, the relationship between the severity in stuttering and activity in the SN is less clear. Possibly, in severely stuttering adults, higher SN activity warrants normal task performance as reflected in the typical behaviour of AWS in the continuous performance task. Alternatively, and more likely, is the view that the task is rather easy to perform and that the brain–behaviour relationship reflects a core neural signature of stuttering, a motor function-related hyperactive SN. A third possible source of the observed relationship could be a variance in iron concentration, which would be unrelated to the task. In general, the brain and in particular the basal ganglia show high levels of iron concentration due to the ubiquitous involvement of iron in biological processes (Gerlach et al. 1994; Berg et al. 2001; Ward et al. 2014) such as the dopamine metabolism (Ben-Shachar et al. 1991). Iron is mainly bound within ferritin, a major iron storage protein, and neuromelanin, a complex polymeric molecule that immobilizes iron. Pigmented neurons of the SN have the highest level of neuromelanin in the brain (Ward et al. 2014). Because iron is paramagnetic, the fMRI signal intensity in the EPI images is rather low in voxels covering the SN. In case the pathomechanism of stuttering is iron-mediated and/or related to different iron concentrations in the SN, the observed correlation could simply mimic a relationship between SN activity and stuttering severity. Iron-sensitive techniques such as transcranial sonography (Gröger and Berg 2012) and iron-sensitive MRI measures (Langkammer et al. 2012, 2016) are suitable methods to find answers to this open question.

A further aspect makes the interpretation of the current finding difficult. The SN is a small brain structure composed of two functionally distinct segments: dopaminergic SNc neurons modulate striatal activity, and GABAergic SNr neurons inhibit thalamic nuclei. Our previous discussion focussed mainly on processes possibly related to a dysregulation of the dopaminergic SNc. This interpretation finds particular support through pharmacological studies, which link stuttering to a hyperdopaminergic state (Alm 2004; Perez and Stoeckle 2016). In this vein, pharmacological agents that block dopamine receptors ameliorate stuttering while dopamine agonists have the opposite effect. However, a complementary view can be drawn when considering the fluency-enhancing effect of the GABA_A_ agonist Pagoclone on stuttering (Maguire et al. 2010). In a large randomized, controlled clinical trial of stuttering, Pagoclone resulted in a fourfold reduction in stuttering, but research for this application was discontinued following disappointing results in Phase II of this clinical trial (Perez and Stoeckle 2016). Pagoclone binds to the benzodiazepine-binding site of human GABA_A_ receptors. Consequently, Pagoclone administration might directly affect activity of the SNr that converges input from the direct and the indirect pathway and provides output from the basal ganglia to the thalamus. Thus, previous pharmacological studies suggest a role of both SNr and SNc to be involved in the pathophysiology of persistent stuttering. Consequently, a positive correlation of SN activity and stuttering severity could reflect an altered activity in the SNr, SNc, or, most likely, in both.

### Altered network dynamics suggest irregular coupling through the D2 receptor-mediated indirect pathway

**Fig. 7:**
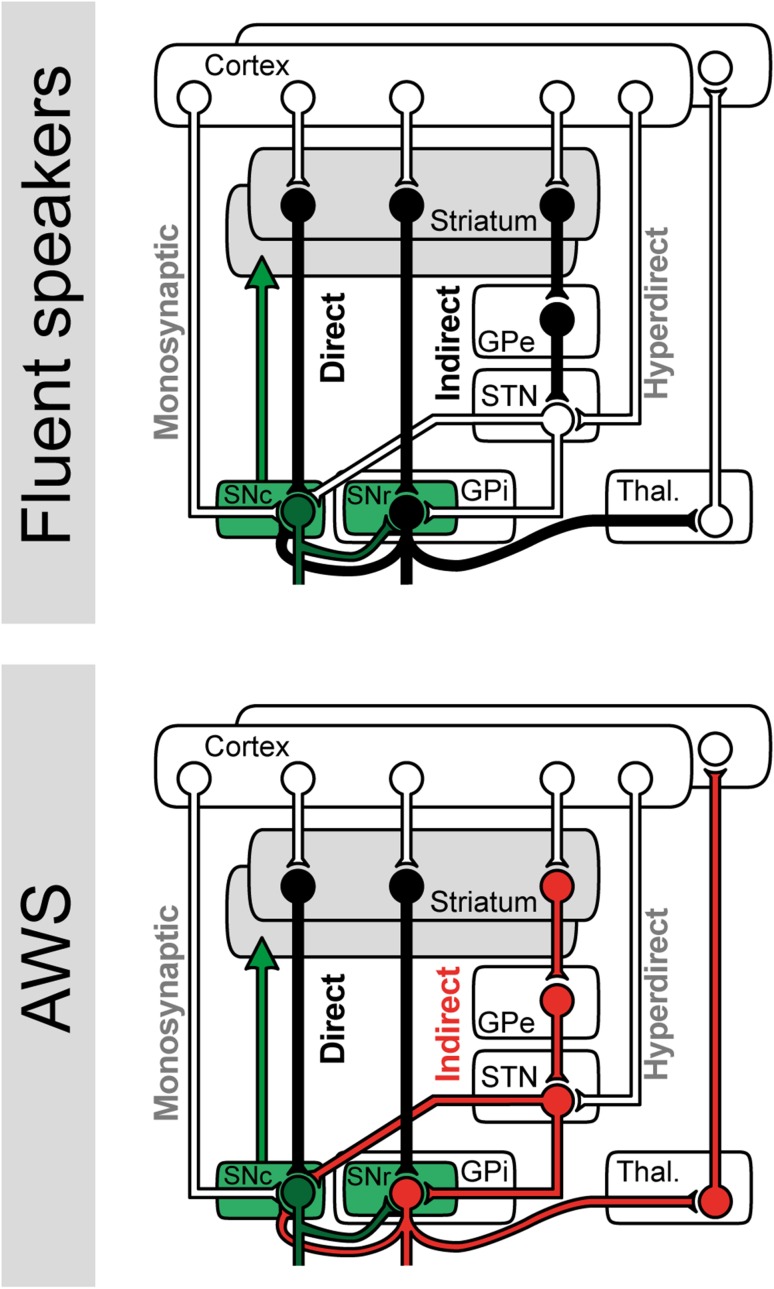
Cortico–basal ganglia–thalamo–cortical loops. The diagram displays a simplified model of the direct, indirect, and hyperdirect pathway. *White* indicates glutamatergic (excitatory) connections, *black* indicates GABAergic (inhibitory) connections, and *green* indicates dopaminergic (modulatory) connections. Adults who stutter exhibited altered network dynamics between cortex and the external segment of the globus pallidus (GPe). For this reason, the GPe and its downstream structures are marked in* red*. Remarkably, the GPe is a principal structure of the indirect pathway

We calculated three psychophysiological interaction analyses to disentangle basal ganglia-mediated network dynamics in the context of persistent developmental stuttering. SN-PPI yielded no significant interaction. GPe-PPI and MD-PPI showed an increased task-related network synchronization in AWS as indicated by a positive PPI. To illustrate dynamic interactions between the basal ganglia, thalamus, and cortex, we provide a simplified schema of involved loops in Fig. 7. According to this diagram, if BOLD reflects the general activity level of a region, then the feed forward branch (GPe-STN-SNr-thalamus-cortex) in the diagram consequently implies the influence of the GPe: increased activity of the GPe should lead to decreased BOLD in the STN and SN but increased BOLD in the thalamus and cortex. In the context of the PPI this means that if the task-related impact of the GPe on cortical activity is strong, activity in both regions should be positively correlated and hence display a positive PPI. A negative PPI, as evident in fluent speakers, does not directly result from such a feed forward chain. It can, however, occur if a more elaborate feedback is involved—mediated by loop structures of the network.

Remarkably, the formation of altered synchronized networks was related to the GPe, a core structure of the indirect pathway. Direct and indirect pathways diverge in the striatum. There, dopamine excites the D1 receptor cells that directly project to the SN, thereby enabling the activation of the intended motor programs. In contrast, striatal dopamine inhibits the D2 receptor cells that project to the GPe. Signal transmission towards the SN is indirect via transsynaptic transmissions through the GPe and STN, aiming at suppressing competing motor programs. Pharmacological intervention with Apomorphine, a mixed D1–D2 receptor agonist, reduces stuttering the same way as the D2 receptor blocker Haloperidol (Burns et al. 1978). It seems that both the stimulating D1 receptors or blocking D2 receptors enhance speech fluency. In accordance with this simplified understanding of basal ganglia circuitries, stimulating D1 receptors facilitates the direct pathway and thus the selection of motor programs, whereas blocking D2 receptors decreases the inhibitory influence of the indirect pathway, thereby, facilitating the focused selection of motor programs. The here observed increased functional coupling between the GPe and the cortex suggests an excessive activity of the indirect pathway and, thus, an increased inhibitory action on the cortex during stuttering, which is in line with previous reports of the fluency-enhancing effect of D2 receptor blockers.

The GPe is an exceptional seed structure for the PPI approach because it represents a structure of the indirect pathway that is not directly influenced by activity of the direct or hyperdirect pathway. It is impossible to resample such an analysis for the other two pathways due to converging input in critical structures. Specifically, the STN receives input from the indirect and the hyperdirect pathway and the SN receives input from all pathways. In this vein, the MD of the thalamus processes the accumulated output of the basal ganglia. Hence, it is not surprising to observe overlapping PPI contrast maps for PPI seeds in the GPe and the MD.

Both PPI analyses, GPe-PPI and MD-PPI, revealed an increased task-related synchronization with activity in the left inferior frontal gyrus, pars opercularis (BA44), adjacent to the left ventral premotor cortex (BA6), and the posterior part of the left inferior frontal sulcus. Previous studies provide accumulating evidence for functional and structural alterations of BA44 in stuttering adults. BOLD responses in the posterior-dorsal part are reduced in AWS during speaking or humming (Neef et al. 2016). AWS lack functional connectivity of left BA44 and premotor regions when speaking or producing non-speech oral motor sounds (Chang et al. 2011), and intrinsic resting-state functional connectivity is reduced in left BA 44 (Lu et al. 2012). Grey matter probability of BA44 is irregular in children who stutter (Chang et al. 2008; Beal et al. 2013, 2015) as well as in adults (Kell et al. 2009; Lu et al. 2012). Altogether, the current results of the functional connectivity analyses advance the view on the role of BA44 in stuttering by showing aberrant dynamics of a response preparation-related network formation via the basal ganglia. Thereby, our data extend recent suggestions from neurocomputational modelling of stuttering. Previously, neurocomputational modelling illustrated that dopaminergic abnormalities as well as a structural disconnection could account for stuttering symptoms by affecting basal ganglia-thalamus-ventral premotor cortex circuits (Civier et al. 2013). Our data suggest an additional involvement of basal ganglia-thalamus-BA44 circuits in the context of response preparation.

### Limitations

In vivo investigations of functional connectivity of the SN are scarce. This is because a precise anatomical localization of this small structure is difficult. The substantia nigra captures a volume of approximately 250 mm^3^ (Keuken et al. 2014; Plantinga et al. 2016). Despite the high in-plane resolution (2 × 2 mm) in the current study, slice thickness was rather coarse (4 mm). Therefore, to minimize partial-volume effects with the signal from the dorsally adjacent STN, we used seed masks of 128 mm^3^ and a small smoothing kernel of 5 mm. Nevertheless, we cannot entirely rule out the contribution of the STN. However, both structures are an integral part of the functional connectome of the brainstem nuclei of the motor network. A recent 7-T fMRI study obtained resting-state fMRI signals of these two structures at a spatial resolution of (1.1 mm)^3^. Delineated Pearson’s correlation-based functional connectomes show that the SN and STN are strongly connected with the thalamus, the dorsal and ventral striatum, pallidum, motor cortex, premotor cortex and SMA, regions of the default mode network, frontal areas such as the frontal pole, SFG and MFG, cerebellum, and limbic regions such as the anterior cingulate and paracingulate areas and the hippocampus (Bianciardi et al. 2016). Thus, it would be plausible to observe overlapping PPI activity in the context of a task requiring response anticipation. PPI studies with seeds in these regions are rather scarce (Köhler et al. 2016) and, to the best of our knowledge, no study exists reporting the seed-based PPI of the STN. Hence, future investigations might help disentangle the subcortical organization of networks and their inference with response anticipation or response inhibition.

Results from connectivity analysis of fMRI data must be regarded with great caution. Here, we asked the question of whether and how functional connectivity with the SN, GPe, and MD is modulated by the preparation of a motor response in AWS. Therefore, we calculated the PPIs. One problem of the PPI approach is that the task regressor may be highly correlated with the physiological regressor. The resulting inflation of the variance is the reason why PPIs are not particularly sensitive, and event-related designs are particularly prone to this problem. An additional confound could be a misspecification of the haemodynamic response model. Such a misspecification could yield correlations that rather reflect activation-induced effects than functional connectivity (Poldrack et al. 2011). In the current study, the regression analysis with the task-based model did not result in differences between groups but the PPI did. An explanation for this could be that the task-based model considered voxels that play a role in a certain condition thus showing the canonical haemodynamic response function when time was locked to the task. In contrast, in the PPI the canonical haemodynamic response function was multiplied by the physiological signal of the seed region, thereby extending the model with a new regressor. Only residuals of the variance that were not explained by the task-based model but fitted this extended model were considered. Voxels of MD and GPe showed task-related co-activity with cortical voxels that was particularly strong during response preparation in AWS as compared to control participants.

## Electronic supplementary material

Below is the link to the electronic supplementary material.

Supplementary material 1 (TIFF 7092 kb)

Supplementary material 2 (TIFF 7878 kb)

Supplementary material 3 (DOCX 38 kb)
