# Supplementary figures and images for: Shifted dynamic interactions between subcortical nuclei and inferior frontal gyri during response preparation in persistent developmental stuttering
Source: Brain Struct Funct. 2017 Jul 24;223(1):165–82. doi: 10.1007/s00429-017-1476-1 (PMC5772149; doi:10.1007/s00429-017-1476-1)

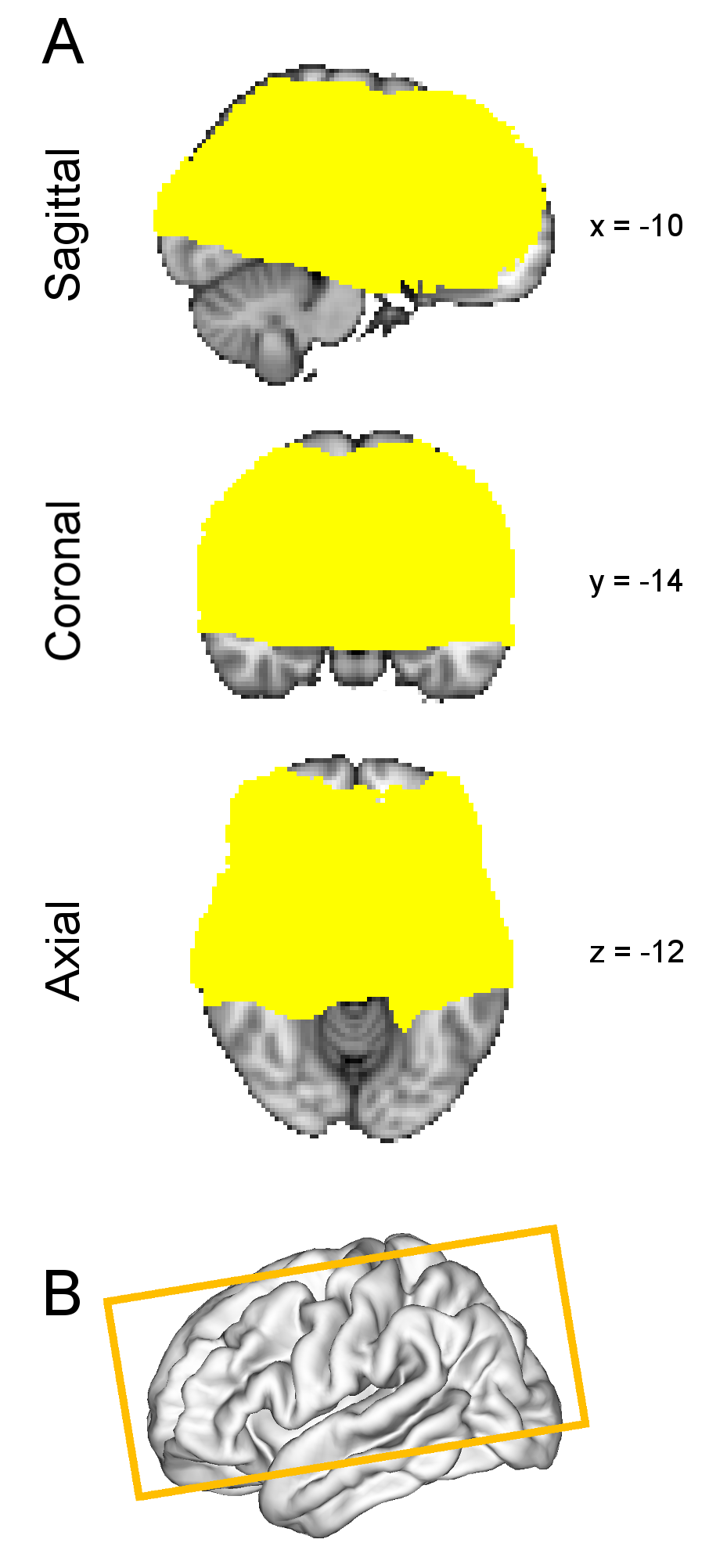

Supplement: Supplementary file 1 — Supplementary material 1 (TIFF 7092 kb) [file 429_2017_1476_MOESM1_ESM.tif]

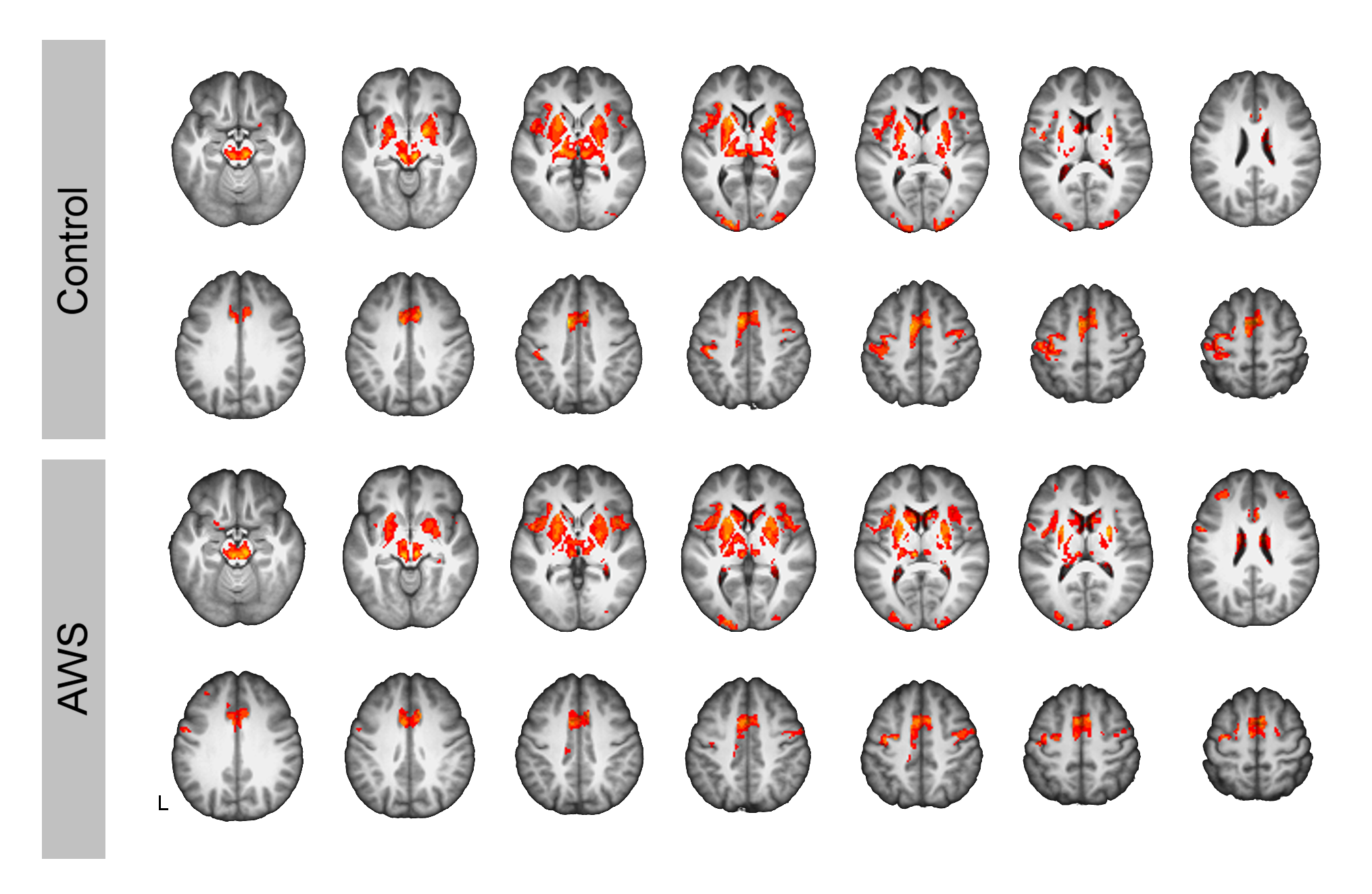

Supplement: Supplementary file 2 — Supplementary material 2 (TIFF 7878 kb) [file 429_2017_1476_MOESM2_ESM.tif]
