# Supplementary material for: Shifted dynamic interactions between subcortical nuclei and inferior frontal gyri during response preparation in persistent developmental stuttering
Source: Brain Struct Funct. 2017 Jul 24;223(1):165–82. doi: 10.1007/s00429-017-1476-1 (PMC5772149; doi:10.1007/s00429-017-1476-1)
Supplement: Supplementary file 3 — Supplementary material 3 (DOCX 38 kb) [file 429_2017_1476_MOESM3_ESM.docx]

**Title: Shifted dynamic interactions between subcortical nuclei and inferior frontal gyri during response anticipation in persistent developmental**

**Authors:** F. Luise Metzger, Tibor Auer, Gunther Helms, Walter Paulus, Jens Frahm, Martin Sommer , and Nicole E. Neef

**Supplementary Materials**

**Supplementary Figure captions:**

**Supplementary Fig. 1 − Group analysis intersection mask.** A) The mask in yellow illustrates how much of the brain was covered after normalization to the MNI standard brain. B) Overlay of the field of view (orange) on the rendered surface of the standard brain.

**Supplementary Fig. 2 − Brain activation during response anticipation.** The upper panel shows

task-positive activity in controls, and the lower panel shows task-positive activity in AWS. Z-statistic

images were thresholded using clusters determined by Z>3.1 and a (corrected) cluster significance threshold of P=0.05 (Worsley 2001).

**Supplementary Tables**

**Supplementary Tab. 1 Participants’ individual characteristics**

|  | **Sex** | **Age** | **LQ** | **Education** | **Mother**  **tongue** | **%ss** | **SSI** | **Severity** |
| --- | --- | --- | --- | --- | --- | --- | --- | --- |
| C1 | m | 26 | 100 | 4 | G |  |  |  |
| C2 | m | 28 | 60 | 4 | G |  |  |  |
| C3 | m | 35 | 89 | 6 | G |  |  |  |
| C4 | f | 25 | 89 | 6 | G |  |  |  |
| C5 | f | 26 | 100 | 5 | G |  |  |  |
| C6 | m | 36 | 100 | 5 | G |  |  |  |
| C7 | m | 42 | 100 | 2 | G |  |  |  |
| C8 | f | 21 | 100 | 2 | G |  |  |  |
| C9 | m | 23 | 100 | 4 | G |  |  |  |
| C10 | f | 23 | 95 | 5 | G |  |  |  |
| C11 | f | 24 | 80 | 4 | G |  |  |  |
| C12 | m | 25 | 90 | 4 | G |  |  |  |
| C13 | m | 25 | 60 | 3 | G |  |  |  |
| C14 | m | 24 | 80 | 4 | G |  |  |  |
| S1 | m | 26 | 100 | 5 | G | 3.10 | 17 | very mild |
| S2 | m | 26 | 100 | 5 | H | 4.10 | 17 | very mild |
| S3 | m | 40 | 100 | 6 | G | 3.10 | 17 | very mild |
| S4 | f | 21 | 88 | 5 | T/G | 3.20 | 27 | moderate |
| S5 | f | 25 | 100 | 3 | G | 27.90 | 48 | very severe |
| S6 | m | 46 | 76 | 2 | G | 4.10 | 23 | mild |
| S7 | m | 38 | 100 | 5 | G | 3.20 | 16 | very mild |
| S8 | f | 36 | 100 | 6 | G | 5.70 | 25 | moderate |
| S9 | m | 20 | 100 | 3 | G | 4.30 | 17 | very mild |
| S10 | m | 38 | 90 | 5 | G | 8.70 | 25 | moderate |
| S11 | m | 24 | 80 | 4 | G | 62.43 | 45 | very severe |
| S12 | m | 24 | 80 | 4 | G | 13.10 | 39 | very severe |
| S13 | f | 23 | 100 | 5 | G | 23.90 | 36 | very severe |

SSI = Stuttering Severity Instrument, third edition; %ss = stuttered syllables occurring per 100 syllables; LQ = laterality quotient, G = German, H = Hungarian, T = Turkish

**Supplementary Tab. 2 Median reaction times and accuracy in the CPT**

|  | **Control** | **AWS** | **Difference** |
| --- | --- | --- | --- |
| *Reaction times* |  |  |  |
| Run 1 | 419.3 (116.6) | 462.2 (135.7) | *p* = 0.449*^M^* |
| Run 2 | 372.9 (52.5) | 406.4 (51.4) | *p* = 0.057*^M^* |
| Run 3 | 354.2 (31.4) | 384.0 (62.7) | *p* = 0.706*^M^* |
| Run 4 | 351.0 (32.8) | 359.7 (38.7) | *p* = 0.449*^M^* |
| *Accuracy* |  |  |  |
| Hit rate | 99.1 (0.6) | 98.4 (1.4) | *p* = 0.185*^U^* |
| False alarm | 0.9 (0.6) | 0.8 (0.6) | *p* = 0.616*^U^* |
| *d*’ | 4.83 (0.31) | 4.78 (0.42) | *p* = 0.830*^U^* |

Group differences were tested by independent-samples median tests*^M^*  or Mann–Whitney *U* tests*^U^*. The Table shows Fisher exact significance (2-sided test).

**Supplementary Tab. 3 Brain activations during response anticipation across all participants (Z > 3.1, p < 0.05)**

| **Region** | **App BA** | **x** | **y** | **z** | **PeakZ** | **Voxels** |
| --- | --- | --- | --- | --- | --- | --- |
| R Red Nucleus |  | 8 | -24 | -6 | 7.00 | 14404 |
| - L Brainstem |  | -6 | -26 | -8 | 6.93 |  |
| - R Putamen |  | 24 | 0 | 12 | 6.84 |  |
| - L Cingulate Gyrus, anterior division |  | -8 | 8 | 40 | 6.80 |  |
| - L Putamen |  | -22 | -6 | 6 | 6.80 |  |
| - R Brainstem |  | 10 | -24 | -16 | 6.78 |  |
| L Occipital Pole |  | -22 | -96 | 4 | 6.29 | 488 |
| - L Lateral Occipital Cortex |  | -32 | -80 | 20 | 4.37 |  |
| R Occipital Pole |  | 20 | -100 | 6 | 5.66 | 451 |
| R Premotor cortex | BA 6 | 42 | -4 | 46 | 5.66 | 281 |
| - R Primary motor cortex | BA 4p | 36 | -16 | 48 | 4.49 |  |
| L Frontal Pole |  | -30 | 44 | 28 | 5.31 | 228 |
| - L Middle Frontal Gyrus |  | -30 | 36 | 26 | 5.05 |  |
| R Frontal Pole |  | 28 | 38 | 24 | 4.78 | 122 |

**Supplementary Tab. 4 Group differences in the functional connectivity of the left MD (Z > 2.3, p < 0.05)**

| **Region** | **App BA** | **x** | **y** | **z** | **PeakZ** | **Voxels** |
| --- | --- | --- | --- | --- | --- | --- |
| L Frontal Pole |  | -38 | 46 | 6 | 3.46 | 694 |
| R Inferior parietal lobule | PFt | 56 | -24 | 36 | 3.5 | 416 |
| - R Inferior parietal lobule | PF/PFcm | 62 | -32 | 20 | 3.15 |  |
| R Cingulate Gyrus, anterior division |  | 8 | 34 | 12 | 3.51 | 382 |
| - R Frontal Pole |  | 22 | 48 | 18 | 2.85 |  |
| L Inferior Frontal Gyrus/Precentral Gyrus | 44/6 | -50 | 4 | 16 | 3.56 | 331 |
| - L Central Opercular Cortex/Insula |  | -32 | 8 | 14 | 3.23 |  |
| - L Insular Cortex |  | -30 | 10 | 10 | 3.19 |  |

**Supplementary Tab. 5 Group differences in the functional connectivity of the left GPe (Z > 2.3, p < 0.05)**

| **Region** | **App BA** | **x** | **y** | **z** | **PeakZ** | **Voxels** |
| --- | --- | --- | --- | --- | --- | --- |
| L Inferior frontal gyrus | 44/45 | -42 | 22 | 18 | 3.63 | 369 |
| - L Inferior frontal gyrus | 45 | -38 | 26 | 18 | 3.57 |  |
| - L Middle frontal gyrus |  | -46 | 34 | 26 | 3.56 |  |
| L Inferior frontal gyrus | 44 | -60 | 12 | 22 | 3.45 | 285 |
| R Inferior frontal gyrus | 45 | 44 | 28 | 18 | 3.7 | 250 |
| - R Inferior frontal gyrus | 44 | 52 | 6 | 18 | 3.53 |  |
